# Study of radiative heat transfer in Ångström- and nanometre-sized gaps

**DOI:** 10.1038/ncomms14479

**Published:** 2017-02-15

**Authors:** Longji Cui, Wonho Jeong, Víctor Fernández-Hurtado, Johannes Feist, Francisco J. García-Vidal, Juan Carlos Cuevas, Edgar Meyhofer, Pramod Reddy

**Affiliations:** 1 Department of Mechanical Engineering, University of Michigan, Ann Arbor, Michigan 48109, USA; 2 Departamento de Física Teórica de la Materia Condensada and Condensed Matter Physics Center (IFIMAC), Universidad Autónoma de Madrid, 28049 Madrid, Spain; 3 Donostia International Physics Center, 20018 Donostia/San Sebastián, Spain; 4 Department of Materials Science and Engineering, University of Michigan, Ann Arbor, Michigan 48109, USA

## Abstract

Radiative heat transfer in Ångström- and nanometre-sized gaps is of great interest because of both its technological importance and open questions regarding the physics of energy transfer in this regime. Here we report studies of radiative heat transfer in few Å to 5 nm gap sizes, performed under ultrahigh vacuum conditions between a Au-coated probe featuring embedded nanoscale thermocouples and a heated planar Au substrate that were both subjected to various surface-cleaning procedures. By drawing on the apparent tunnelling barrier height as a signature of cleanliness, we found that upon systematically cleaning via a plasma or locally pushing the tip into the substrate by a few nanometres, the observed radiative conductances decreased from unexpectedly large values to extremely small ones—below the detection limit of our probe—as expected from our computational results. Our results show that it is possible to avoid the confounding effects of surface contamination and systematically study thermal radiation in Ångström- and nanometre-sized gaps.

## Abstract (web summary)

Here, Cui *et al*. report radiative heat transfer in few Ångström to 5 nm gap sizes, between a gold-coated probe and a heated planar gold substrate subjected to various surface cleaning procedures. They found that insufficiently cleaned probes and substrates led to unexpectedly large radiative thermal conductances.

Radiative heat transfer at the nanoscale is of fundamental significance^1^,^2^,^3^,^4^,^5^,^6^,^7^,^8^,^9^,^10^, and holds promise to improve a variety of technologies ranging from thermophotovoltaic energy conversion^11^,^12^,^13^,^14^,^15^,^16^,^17^, non-invasive thermal imaging^18^ to heat-assisted magnetic recording^19^ and nanolithography^20^. It has been predicted^2^,^21^, based on the theoretical framework of fluctuational electrodynamics^22^, that the radiative heat flux can exceed the Planck’s blackbody limit by several orders of magnitude when two surfaces are brought into the near-field (gap size smaller than the Wien’s wavelength, ∼10 μm at room temperature). With the advancement of new experimental techniques over the past decade, a number of experimental studies^1^,^3^,^23^,^24^,^25^,^26^,^27^,^28^,^29^ have demonstrated super-Planckian thermal transport for gap sizes ranging from hundreds of nanometres to as small as 2–3 nm. Generally, the results from these measurements were found to be in good agreement with predictions based on fluctuational electrodynamics for a broad range of materials and geometries.

In spite of the important progress described above, there remains significant disagreement in the literature about radiative heat transfer in the extreme near field (gap size <10 nm). Specifically, measurements^30^,^31^,^32^ for two gold (Au)-coated surfaces with gap size in the range of ∼0.2–10 nm have suggested an extraordinarily large near-field enhancement—over 3 orders of magnitude larger than the predictions from conventional fluctuational electrodynamics^22^,^33^. These surprising results question the validity of current theories of heat transfer for these small gaps. Researchers have explored the possibility of reconciling the experimental data with computations, by relaxing the local approximation^21^ that is often employed in calculations of near-field radiative heat transfer; however, such investigations^34^,^35^ suggest that the inclusion of nonlocal effects leads to relatively modest changes of heat fluxes in the extreme near-field (down to gap sizes of a few Å). The contribution of phonons to thermal transport across vacuum gaps has also been investigated^36^,^37^,^38^,^39^,^40^. For example, a recent computational analysis^41^ of radiative heat transfer between plane-parallel surfaces of NaCl, based on microscopic Maxwell’s equations, has shown that deviations from the predictions of fluctuational electrodynamics (for example, via acoustic phonon tunnelling), occur only for gap sizes <0.5 nm. In particular, at a gap size of ∼3 Å the phononic contribution to the total thermal conductance was found to be approximately three times higher than its photonic counterpart. Further, recent experimental work by us^1^, performed using cantilevers with embedded thermocouples, quantitatively measured the extreme near-field radiative heat transfer down to gap sizes of ∼2 nm for polar dielectrics and ∼3 nm for metallic materials. In contrast to the tunnelling current-based measurements of gap size employed by others^30^,^31^,^32^, our measurements were performed using compliant cantilevers that enabled direct detection of mechanical contact ensuring that we performed measurements only under conditions where a vacuum gap was present between the surfaces that were being studied. However, because of the compliance of the cantilevers the smallest gaps that could be accessed were in the 2–3 nm range. Although our work found excellent agreement with the predictions of fluctuational electrodynamics down to gap sizes of 2–3 nm (deviations were <15%), it remained unclear whether large discrepancies between theory and experiment, as reported by recent experimental work^30^,^31^,^32^, arise in gaps of a few Ångströms.

Here we explore radiative heat transfer in Ångström- and nanometre-sized gaps between a Au-coated scanning thermal microscopy probe (SThM) and a heated planar Au substrate in an ultrahigh vacuum (UHV) environment. We first describe the SThM set-up and the experimental strategy employed in our measurements. Subsequently, we present experimental results for probes and substrates subject to both *ex situ* (plasma-cleaning) and *in situ* (controlled crashing) cleaning procedures. We show that insufficiently cleaned probes and substrates lead to unexpectedly large thermal conductances, vastly exceeding computational predictions for radiative conductances and feature small apparent tunnelling barrier heights (∼1 eV), suggesting the presence of surface contamination that provides a parasitic path for heat transfer via conduction. We also show that systematic cleaning of the probe and substrate surfaces increases the apparent barrier height to values as large as 2.5 eV and the measured conductances reduce to considerably small values that are below the detection limit of our probes as expected from our computations.

## Results

### Experimental set-up and strategy

**Figure 1:**
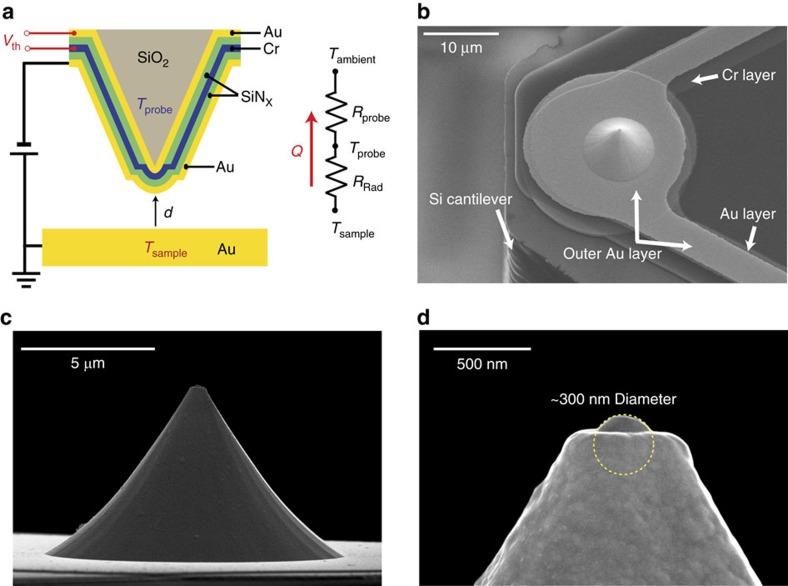
Experimental set-up and SEM images of the SThM probes and gold-coated tips. (**a**) Schematic of the experimental set-up, in which a Au-coated SThM probe (cross-sectional view) is brought into close proximity of a heated Au substrate. The tunnelling current across the nanogap is monitored by applying d.c. or a.c. voltages. Simultaneously, the thermoelectric voltage (*V*_th_) generated by the Au–Cr thermocouple is recorded to monitor the temperature of the probe’s tip. The diagram on the right shows the thermal resistance network representing the heat flow from the substrate, through the nanogap, to the probe. (**b**) SEM image showing the top view of a SThM probe. (**c**,**d**) SEM images of the probe and its tip, which has a diameter of ∼300 nm.

We experimentally studied radiative heat transfer using custom-fabricated SThM probes (Fig. 1, see Supplementary Note 1 and Supplementary Fig. 1 for details of probe fabrication). A key characteristic of these SThM probes is their high stiffness (∼10^4^ N m^−1^, see Supplementary Note 2 and Supplementary Fig. 2), which is in strong contrast to the compliant probes (∼5 N m^−1^; ref. 42) used in our recent work^43^ and enables measurements down to Å-sized gaps. These SThM probes also feature an integrated Au–Cr thermocouple (Fig. 1a), located in close proximity to the probe tip to form a sensitive thermal sensor^42^,^44^ with a very small thermal time constant (∼10 μs; ref. 44) and a spherical Au tip with a diameter of ∼300 nm. In order to study radiative heat transfer in nanoscale gaps we mounted the SThM probes into an UHV scanning probe instrument and recorded the change in the thermovoltage across the thermocouple. In our measurements, a SThM probe, which was initially at a temperature *T*_probe_, was displaced at a constant speed (0.1 nm s^−1^) by piezoelectric actuation towards a planar Au sample at an elevated temperature *T*_sample_. The applied temperature differential 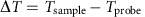 resulted in a radiative heat flow (*Q*) from the Au sample to the probe and elevates the probe temperature to 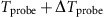. From the thermal resistance network shown in Fig. 1a the near-field radiative thermal conductance (*G*_Rad_=(*R*_Rad_)^−1^) is determined to be 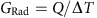, where *Q* can be related to the thermal resistance of the probe (*R*_probe_) by 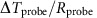. We note that the resistance of the probe *R*_probe_ was independently measured (see Supplementary Note 3 and Supplementary Fig. 3) to be 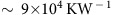, following an approach developed by us recently^43^. Further, 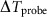 was related to the thermovoltage (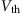) generated across the Au–Cr thermocouple by 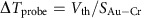, where 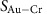
=16.3 μV K^−1^ is the Seebeck coefficient of the thermocouple^44^. We determined ‘contact’ of the probe with the sample by applying a small AC bias of 1 mV at 10 kHz across the tip and the sample and monitoring the amplitude of the tunnelling current at the same frequency using a lock-in amplifier. For the purposes of this experiment we defined contact as the situation when the tunnelling current exceeds an amplitude of 10 nA (see Methods), corresponding to a tunnelling resistance of ∼0.1 MΩ, which is known to correspond to a very small gap size^45^,^46^ (1–2 Å). We note that the effect of Joule heating due to the tunnelling current is negligible (∼5 pW), as it is much smaller than the radiative heat flux (see below).

### Experimental results

**Figure 2 f2:**
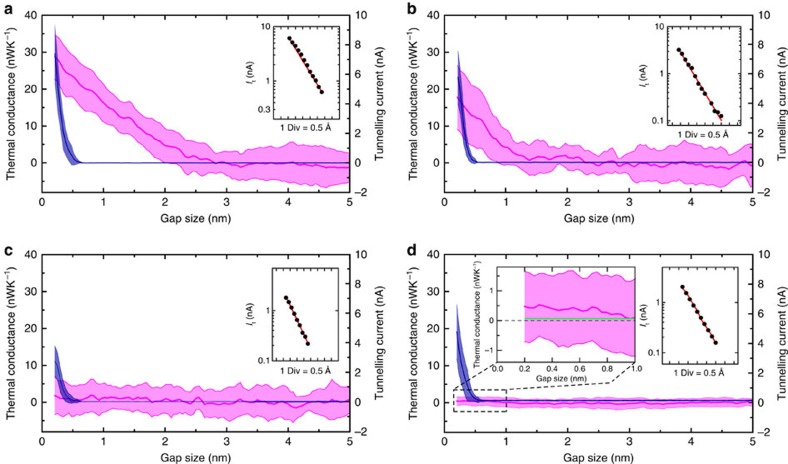
Measured gap size-dependent thermal conductance and tunnelling current. Each thermal conductance (pink) and tunnelling current (blue) curve is averaged over 15 repeated measurements. The shaded region represents the s.d. The first three panels show representative experimental results from (**a**) organic solvent-cleaned, (**b**) oxygen plasma-cleaned and (**c**) repeated oxygen plasma-cleaned probes. The gold sample is heated while the probe is maintained at a lower temperature to create a temperature difference Δ*T=*40 K. (**d**) The measurement results obtained in experiments where a large temperature differential (Δ*T*) of 130 K was applied. The thermal conductance data in the subnanometre region is shown on an expanded scale in the inset to facilitate visualization. The green line in the inset panel corresponds to the near-field radiative thermal conductance calculated from fluctuational electrodynamics. Further, the measured tunnelling currents versus displacement are shown in insets for each of the plots and were used in the analysis of the apparent tunnelling barrier height *φ*_ap_. The estimated values of *φ*_ap_ are 1.1, 1.6, 1.7 and 1.9 eV for **a**–**d**, respectively.

In the first set of experiments a probe, thoroughly cleaned in acetone to remove potential contaminants and residue from fabrication and handling, was loaded into a UHV scanning probe microscope along with a template-stripped Au surface (immediately after stripping the sample, see Supplementary Note 4 and Supplementary Fig. 4 for details of surface characterization of scanning probes and the Au surface) to study near-field radiative heat transfer (see Methods for a discussion of the template-stripping procedure). In this experiment the sample temperature (*T*_sample_) was chosen to be 343 K, while the probe was held at a lower temperature (*T*_probe_=303 K), thus establishing a temperature differential (Δ*T*) of 40 K. This temperature differential was chosen to be significantly smaller than the average temperature of the sample and the probe (*T*_sample_+*T*_probe_)/2 to ensure that experiments were conducted in the linear response regime. We note that applying larger temperature differentials (for example, 100 K) does not markedly affect the estimated conductances as discussed in more detail below [Fig f2][Fig f3][Fig f4](see Fig. 5b). The measurements were performed at various locations on the template-stripped Au sample to evaluate any possible surface inhomogeneity effects. Figure 2 shows the measured radiative thermal conductance (pink) and tunnelling current (blue) from 15 independent measurements as the gap size between the probe and the sample is reduced at a constant speed (∼0.1 nm s^−1^). The solid lines represent the mean value of the data, whereas the transparent colour represents the standard deviation (s.d.). It can be seen from the data that the observed thermal conductance begins to increase monotonically as the gap size is reduced below ∼2.5 nm and reaches a value as large as ∼30 nWK^−1^ at the smallest gap size. This behaviour is similar to what was reported in recent papers^31^,^32^. As a comparison, in ref. 31, the measured heat flux begins to monotonically increase below gaps of ∼4.5 nm, whereas in ref. 32 for a probe similar to that used in ref. 31, the monotonic increase with declining gap size starts at ∼6 nm, and the thermal conductance at the smallest gap is reported to be ∼3 nWK^−1^—a value smaller than that observed by us, possibly because of the smaller tip size of the probes employed in ref. 32 (our probe’s diameter is approximately five times larger). This experimentally observed conductance (∼30 nWK^−1^) for our solvent-cleaned probes is almost three orders of magnitude larger than that predicted by calculations performed using fluctuational electrodynamics (see Fig. 5, calculation details explained later).

A possible explanation for the large thermal conductance observed in our first experiments with solvent-cleaned probes is the presence of contaminants that may bridge the tip and the sample before the Au on the tip contacts the Au atoms on the sample, thus providing a pathway for heat transfer via conduction. One may hypothesize that such contamination arises from imperfect removal of any molecules bound to the SThM probe during fabrication, contamination during storage and handling, or due to recontamination of the tip due to diffusion of molecules present elsewhere on the sample or the tip. Given the small size of the SThM tip (diameter of ∼300 nm), a direct characterization of its surface is challenging. Therefore, as a first test of our hypothesis that the sample is contaminated we analysed the tunnelling current curves following a procedure reported elsewhere^47^,^48^. Specifically, we fitted the tunnelling current data to a tunnelling barrier model to obtain the apparent barrier height (*φ*_ap_, in eV) from 
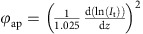
, where *z* is the gap size in Å and *I*_t_ is the tunnelling current. From the slope of the tunnelling current shown in Fig. 2a we obtained a value for *φ*_ap_ of 1.1 eV. This low barrier height is in contrast to the large (∼4.7±1 eV) barrier heights^47^ expected for ultraclean Au surfaces and is indeed consistent with the presence of surface contamination^47^,^48^. We note that careful analysis of tunnelling current curves presented in previous studies^32^ also suggested low barrier heights (<1 eV), indicating the possibility of contamination.

In order to explore the feasibility of reducing or eliminating surface contamination effects, we employed oxygen plasma-based techniques^49^ for cleaning the probe (see Methods). Subsequently, we repeated the conductance measurements following an approach identical to that used in obtaining the data in Fig. 2a. The data obtained from this experiment are shown in Fig. 2b, in which we can clearly see that the thermal conductance is reduced by a factor of 2 and the thermal conductance starts to monotonically increase from a smaller gap size of ∼1 nm. Since this measurement employed the same probe, sample and experimental procedures as that in Fig. 2a, we conclude that surface contaminants are the most probable reason for the observed spurious thermal conductances. This conclusion is further supported by our analysis of the tunnelling current data (obtained from the cleaned sample and tip), which yielded a *φ*_ap_ of 1.6 eV, a value significantly larger than that obtained for the data in Fig. 2a.

Upon reducing the effect of potential surface contaminants, we repeated the oxygen plasma-cleaning process (three times for both the probe and the sample) to evaluate whether the cleanliness can be further improved. Data obtained from experiments performed after these additional cleaning steps are shown in Fig. 2c. It can be seen that there is no discernible increase in thermal conductance until the probe contacts the sample. Further, the apparent barrier height was further increased to ∼1.7 eV, reflecting the increased cleanliness of the surfaces. The noise floor in thermal conductance (root mean square value ∼3 nWK^−1^) measurements has significant contributions from low-frequency noise associated with temperature drift of the ambient (see Supplementary Note 5 and Supplementary Figs 5 and 6 for details) and from Johnson noise of the thermocouple, whose electrical resistance is ∼5 kΩ. The data in Fig. 2c (corresponding to the solid line) show that the thermal conductance is less than 2.5 nWK^−1^ for subnanometre gaps. To get a more refined estimate of this conductance, we increased the temperature differential to 130 K by increasing the sample temperature to 445 K (the probe temperature increases to 315 K). Such increased temperature differentials resulted in an enhanced signal-to-noise ratio as the noise remains (largely) unchanged, whereas the signal increases, to first order, proportionally to the applied temperature differential. This larger temperature differential leads to deviations from the linear response regime; however, the expected deviations in the thermal conductance are small (see inset of Fig. 5b). Further, application of this larger temperature differential enables a more direct comparison with past experiments^30^,^31^,^32^ where similarly large temperature differentials were applied. Data obtained from these experiments (Fig. 2d) show a significantly reduced noise floor (∼900 pWK^−1^, RMS) and the mean from 15 different experiments (solid pink line in the inset) shows that the maximum possible thermal conductance at the smallest gaps (∼2 Å) is ∼0.5 nWK^−1^ —a value much smaller than that reported in previous works^30^,^32^.

**Figure 3 f3:**
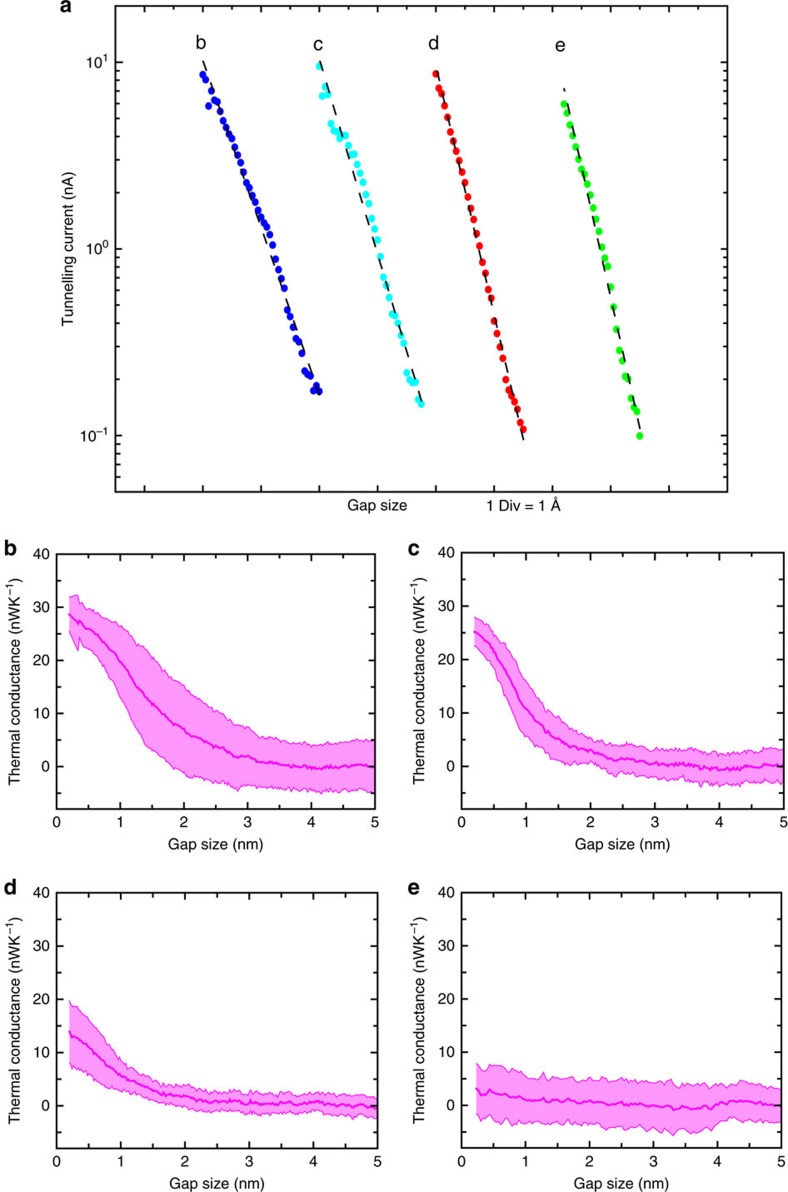
Apparent tunnelling barrier and thermal conductance by controlled-crashing cleaning. Solvent and plasma-cleaned probes were used in experiments where the probe was intentionally indented into the substrate by a few nanometres to create a direct point contact between the probe and the sample. This procedure resulted in gaps that featured larger apparent tunnelling barrier heights. Specifically, *φ*_ap_ was found from tunnelling current versus displacement curves (**a**) to monotonically increase from 1 eV (dark blue dots, for the probe in initial condition) to 1.4 eV, to 2.2 eV and finally to 2.5 eV in consecutive experiments where the tip was displaced into the substrate by 1 nm (light blue dots), 2 nm (red dots) and 5 nm (green dots), respectively. The thermal conductance corresponding to each of these scenarios is shown in **b**–**e**. It can be seen that the apparent near-field thermal conductance is systematically reduced as the apparent tunnelling barrier height increases.

In addition to the approaches described above, we further explored the feasibility of cleaning the tip and the sample locally by ‘controlled crashing/displacing’ of the tip into the sample by 1–5 nm. This *in situ* cleaning method has been reported previously to be effective in locally cleaning surfaces^47^,^48^,^50^. We used a probe that was initially cleaned with liquid solvents and oxygen plasma procedures but which still showed spurious measured thermal conductances, possibly because of partial success in our cleaning procedure or recontamination. The blue line in Fig. 3a shows the measured gap-dependent tunnelling current (before performing *in situ* cleaning) and features an apparent tunnelling barrier of ∼1.0 eV. Further, the corresponding thermal measurement (Fig. 3b) reveals large thermal conductances (∼20–30 nWK^−1^) in gaps below 1 nm, consistent with the presence of contamination. Following the initial characterization of thermal conductance we performed an experiment where the probe was displaced into the substrate by 1 nm after an electrical conductance of 1 *G*_0_ (*G*_0_=2*e*^2^/*h*, the quantum of electrical conductance, corresponding to a single-atom contact) was established. After the experiment the apparent barrier height had increased to ∼1.4 eV (Fig. 3a, light blue) and the thermal conductance in subnanometre gaps decreased modestly to a value of ∼25 nWK^−1^ (Fig. 3c). In order to explore the feasibility of achieving greater cleanliness we performed two additional experiments where we made more aggressive indentations (that is, displacement of the tip into the substrate by 2 and 5 nm after making atomic contact). After the 2 nm displacement a much larger apparent barrier height ∼2.2 eV was measured (Fig. 3a, red). However, the thermal conductance was reduced only modestly to ∼10 nWK^−1^. This incomplete cleaning is possibly due to the local nature of the cleaning procedure, which results in removal of contaminants only directly under the tip while leaving the surrounding region unaffected. However, the data from the 5 nm displacement experiments showed a significantly reduced thermal conductance of ∼2 nWK^−1^ (Fig. 3c) in subnanometre gaps as limited by the noise floor of our technique (the applied temperature differential was 40 K), whereas the barrier height was found to increase to 2.5 eV (Fig. 3a, green). These experiments show that the effect of contamination can be systematically reduced by locally crashing the tip into the sample. We note that the tip shape of the probes subjected to the *in situ* cleaning method does not undergo any observable change (as observed from scanning electron microscopy (SEM) images of the probes, see Supplementary Note 6 and Supplementary Fig. 7). Therefore, we conclude that the surface changes that may occur because of the exchange of Au atoms between the tip and the substrate are microscopic in nature. We show in Fig. 5 that such small variations in the surface roughness are not expected to result in significant changes in near-field radiative heat transfer and hence cannot be the source of the observed decrease in thermal conductance.

**Figure 4 f4:**
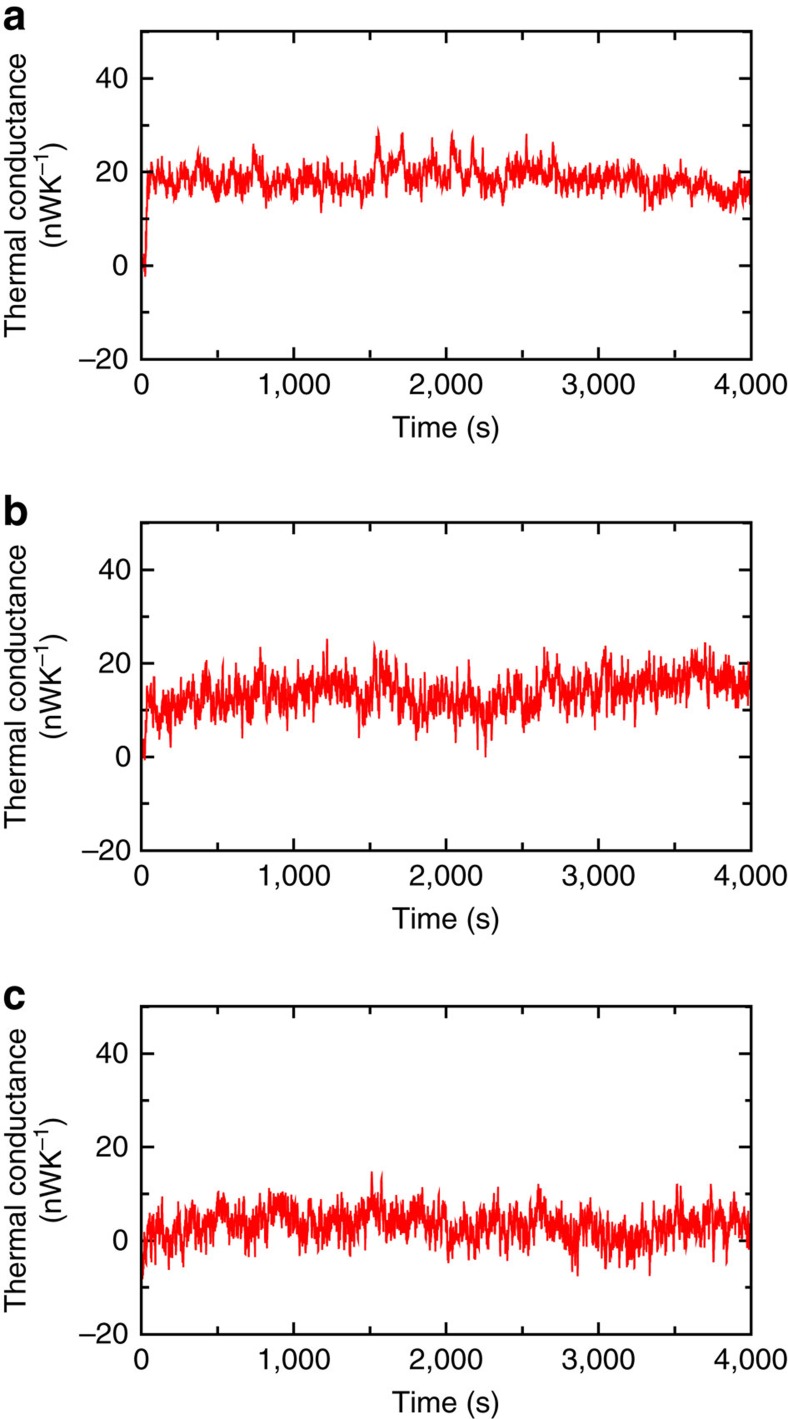
Time-dependent thermal conductance for probes subjected to different cleaning procedures. (**a**–**c**) Thermal conductance as a function of time for probes subjected to the cleaning procedures as described for Fig. 2a–c, respectively.

Finally, we performed one more set of experiments where we explored how robust the cleaning procedures are and if the tip and the sample can be potentially re-contaminated. Specifically, we placed the probe at a constant separation from the sample by maintaining the tunnelling current at 1 nA (50 mV d.c. bias, corresponding to a gap size of ∼5–7 Å) via feedback and continuously measured the thermal conductance of the gap as a function of time. For a probe and sample that were as clean as those used in obtaining the data shown in Fig. 2a (they were subjected to the same cleaning procedures and had an apparent barrier height of ∼1 eV) the thermal conductance was relatively unaffected (see Fig. 4a) over a long period of time (∼1 h). Similar measurements were also performed on probes and samples that are expected to have cleanliness similar to those used in obtaining the data shown in Fig. 2b,c (as reflected from their apparent barrier heights). The data obtained from these experiments are shown in Fig. 4b,c, respectively. It can be seen that the thermal conductance in these experiments is also relatively stable. The low-frequency noise seen in these plots is most probably due to ambient temperature drift and is quantified in the SI. Taken together, these data suggest that the probability of contamination is relatively low for well-cleaned probes in an UHV environment.

### Computational results

**Figure 5:**
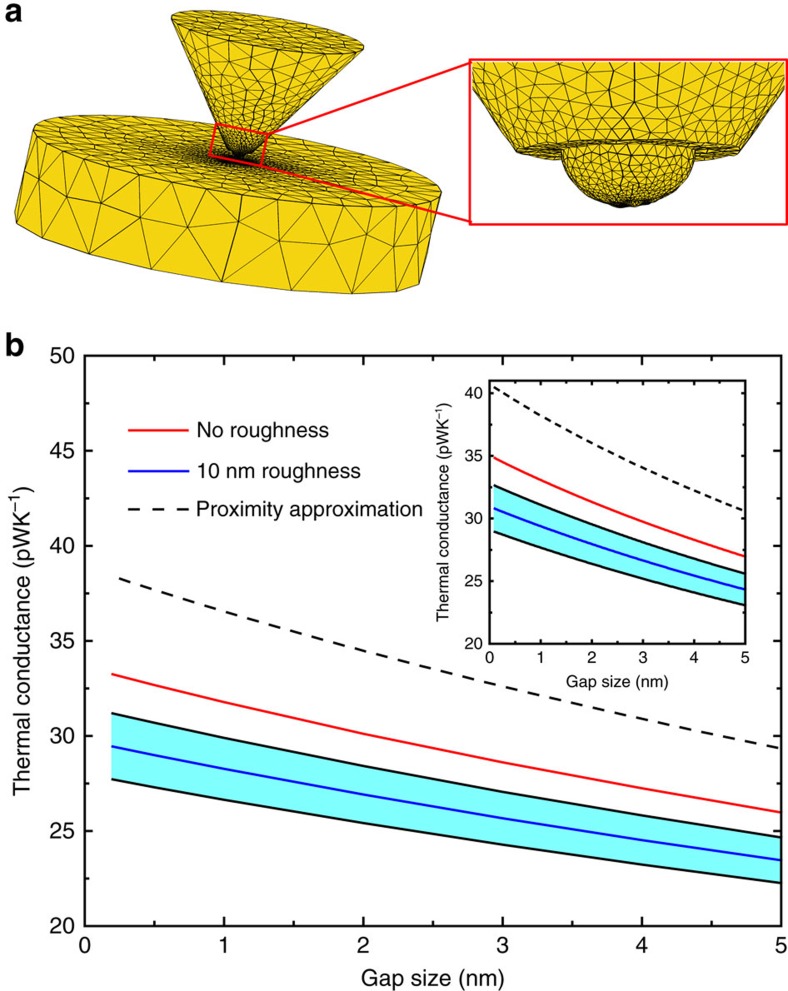
Computational prediction of the radiative thermal conductance. (**a**) Tip-substrate geometry employed in our numerical simulations. Following the SEM images of our thermal probes, the tip was modelled as an irregular cone that ends in a hemisphere, while the substrate was modelled as a thick disk. The height of the cone was chosen to be 3 μm and the radius of its base was 1.9 μm. The radius of the disk was 4 μm and its thickness was 2 μm. The solid black lines depict the triangular mesh employed in the boundary element method (BEM) calculations. The right inset shows a blow-up of the tip apex region. (**b**) The computed total radiative thermal conductance as a function of the gap size between the Au tip and substrate. The red solid line corresponds to the ideal tip (no roughness) and the blue line to the average obtained for 15 different tips with stochastically chosen roughness profiles (RMS roughness ∼2–3 nm), while the shaded region indicate the s.d. The black dashed line is the computed thermal conductance from the proximity approximation for the case of no roughness. The tip diameter in these calculations is 300 nm, while the temperature of the probe and substrate were chosen to be 303 and 343 K, respectively. Inset, similar to the main panel except that the probe and substrate temperatures are 315 and 445 K, respectively.

In order to determine whether our experimental results can be explained in the light of established NFRHT theories, we used the fluctuating-surface-current formulation of the radiative heat transfer problem^33^,^51^. In practice, we made use of a combination of this formulation with the well-established boundary element method, as implemented in the SCUFF-EM solver^52^. This combination allows us to describe the radiative heat transfer between bodies of arbitrary shape and provides numerically exact results within the framework of fluctuational electrodynamics in the local approximation (in which the dielectric functions of the materials are assumed to depend only on frequency). In particular, this approach was successfully employed by us^1^ to describe the NFRHT between compliant atomic force microscope-based scanning thermal probes and substrates coated with metals/dielectrics for separations (or gaps) down to 2–3 nm. In order to simulate our experiments as accurately as possible, we considered tip-substrate geometries like the one shown in Fig. 5a. Here we followed the SEM images of our thermal probes and modelled the gold tip with an irregular conical shape that ends in a hemispherical cap of radius 150 nm, while the substrate was modelled by a thick disk whose dimensions have been carefully chosen to avoid any finite-size effects. Following the approach taken in our previous work^1^, we also simulated the roughness of our gold tips by including a random Gaussian noise in the profile of the tip apex (inset of Fig. 5a). To be precise, the maximum protrusion height of our roughness is 10 nm and the correlation length between protrusions is 17 nm. To evaluate the impact of the roughness in the radiative heat transfer in our system, we simulated an ensemble of 15 tips with different roughness profiles. We emphasize that the only input information in our simulations, apart from the geometry, is the frequency-dependent dielectric function of gold that we obtained from published work^1^.

The results of the simulations for the total radiative thermal conductance between the gold tip and the gold substrate are shown in Fig. 5b for gap sizes from 1 Å to 5 nm. This figure displays the results for an ideal tip (no roughness) and for 15 tips featuring roughness (both mean value and s.d.). It can be seen that the tip roughness has no major impact on the thermal conductance. More importantly, the thermal conductance for the smallest gap size is of the order of 30 pWK^−1^, which is ∼30 times smaller than the noise floor (∼900 pWK^−1^, RMS) in our large bias measurements (see Fig. 2d). The results obtained by using the proximity approximation^53^ are also shown in Fig. 5b and are found to show similar trends (that is, increase in conductance with reducing gap size) as our exact simulations but fail to accurately reproduce the simulation results. These calculations support our conclusion that the large signals observed in our experiments before the cleaning procedure cannot be explained in terms of radiative heat transfer. For completeness, we note that the slow decay of the thermal conductance with gap size (Fig. 5b) is the characteristic of metals. This is due to the fact that radiative heat transfer is dominated by evanescent (in the vacuum gap) transverse electric modes resulting from total internal reflection, the contribution of which saturates at single-nanometre gaps^1^,^34^. In addition, the role of nonlocal effects in the dielectric function of gold has been studied^34^ in the context of radiative heat transfer and has been shown to be very small for the gap sizes explored in our work.

## Discussion

An interesting question pertains to the gap size at which fluctuational electrodynamics fails to describe radiative heat transfer. First, we note that, while some deviations from computational predictions can be seen in the inset of Fig. 2d for subnanometre-sized gaps these deviations are within the noise floor of our measurement technique, making it hard to draw any robust conclusions about the failure of current theories. We note that for small gap sizes electrons can also make significant contributions to heat transfer. For Au electrodes, the electronic contribution to thermal conductance (*G*_th, electronic_) can be readily computed using the Wiedemann–Franz law that relates *G*_th, electronic_, to the electrical conductance *G*_e_, by *G*_th, electronic_*=L*_0_*TG*_e_, where *L*_0_=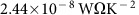 is the Lorentz number and *T* is the absolute temperature. Since the measured electrical conductance, corresponding to the smallest gap sizes in our experiments, is ∼0.1 *G*_0_ we estimate *G*_th, electronic_ to be ∼60 pWK^−1^, which is also below our noise floor. Our experiments show that further improvements in the resolution of experimental techniques are necessary to unambiguously ascertain whether and at what gap sizes current theories fail to describe near-field thermal radiation.

To summarize, we report measurements of extreme near-field radiative heat transfer at gap sizes ranging from a few Å to 5 nm. Our results suggest that past reports^30^,^31^,^32^ of large deviations from the predictions of fluctuational electrodynamics are probably because of surface contamination effects. We also demonstrate, from measurements of apparent tunnelling barrier heights, that such deviations can be systematically attenuated by carefully cleaning the surfaces as indicated by an increase in the apparent barrier height. In contrast to previous studies^30^,^31^,^32^, which observed both conductances as large as 3 nWK^−1^ (three to four orders larger than the predictions of fluctuational electrodynamics) and conductance enhancements beginning at gap sizes as large as 4–5 nm, our results (Fig. 2d) suggest that deviations, if any, are in the subnanometre regime and are much smaller in magnitude (∼0.5 nWK^−1^, based on the observed mean value). These deviations in subnanometre-sized gaps could potentially result from monolayer-level contaminations that may still be present on our surfaces and cannot be detected from our probes. Further, this work highlights the need for the development of probes, for example, based on the approach leveraged in our previous work^1^,^54^, that can accurately resolve the small heat fluxes expected for Au surfaces at subnanometre gaps while independently quantifying the gap size, surface roughness and interaction forces with the substrate. Such approaches are crucial for drawing careful conclusions about extreme near-field radiation. The insights obtained from this study will be critical for the development of high-sensitivity near-field measurements, and future technologies that leverage nanoscale radiative heat transfer.

## Methods

### Description of experimental conditions

In order to minimize perturbations of the gap size due to vibrations, thermally induced fluctuations and due to electrostatic or Van der Waals forces the probe was designed to have a very high stiffness (∼10^4^ Nm^−1^, see Supplementary Note 2). Further, the scanning probe microscope (RHK UHV 7500) was housed in an ultralow-noise facility with high vibration-isolation capability (NIST-A) and low-temperature drift (<0.1 °C over 24 h). Moreover, to exclusively measure near-field radiative heat transfer and attenuate the contribution of air and water molecules in the nanogap to negligible levels, experiments were performed in an UHV environment (<3 × 10^−9^ torr).

### Template-stripping procedure

Flat and clean Au substrates were created using a template-stripping technique. Briefly, 100 nm of gold was deposited on a pristine Si wafer. Subsequently, a low-viscosity high-temperature epoxy (Epotek 377) was applied evenly to the gold-coated Si wafer to glue (attach) clean 7 mm × 7 mm glass pieces to the top of the wafer. The wafer was cured at ∼150 °C for an hour. Right before the experiment the glass piece with the deposited gold layer was cleaved off from the wafer in a nitrogen-filled glove box, resulting in a fresh, template-stripped gold sample.

### Surface-cleaning procedures

To systematically clean the scanning probe and get a clean gold surface, the nanofabricated probes were sonicated in acetone, then isopropyl alcohol and subsequently in deionized water. Each cleaning step of this sequence was applied for ∼10 min to remove organic contamination from the probe. Subsequently, the probe was dried and subjected to oxygen plasma-cleaning cycles (300 W, 120 mTorr, 5 min). Oxygen plasma-cleaning cycles were repeated as described in the main text. For all our experiments, clean gold samples and probes were always placed in a nitrogen gas environment to avoid direct exposure to the ambient. In our experiments, exposure of the probe and sample surfaces to the ambient environment for a few minutes is found to be sufficient to re-contaminate the cleaned surfaces, resulting in spurious thermal conductances.

### Determination of gap size

In our experiments the SThM probe was displaced towards the substrate at a constant speed (0.1 nm s^−1^), by a piezoelectric actuator, starting from an initial distance of ∼5 nm until ‘contact’ was established with the substrate. We define ‘contact’ as the situation where a tunnelling gap resistance *R*=0.1 MΩ is reached, which based on the measured apparent tunnelling barrier *φ*_ap_ (∼1–3 eV), corresponds to an estimated gap size of ∼1.3–2.3 Å. To validate this estimation, we have also performed experiments to measure the actual distance between the defined ‘contact’ (0.1 *G*_0_) and the Au–Au atomic contact (1 *G*_0_) and found that it was in good agreement with the extrapolated distance (see Supplementary Note 7 and Supplementary Fig. 8). To account for this separation all data presented in Figs 2 and 3 feature a minimum gap size of 2 Å.

### Data availability

The data that support the findings of this study are available from the corresponding authors upon request.

## Additional information

**How to cite this article:** Cui, L. *et al*. Study of radiative heat transfer in Ångström- and nanometre-sized gaps. *Nat. Commun.* **8,** 14479 doi: 10.1038/ncomms14479 (2017).

Publisher’s note: Springer Nature remains neutral with regard to jurisdictional claims in published maps and institutional affiliations.

## Supplementary Material

Supplementary Information Supplementary Figures, Supplementary Notes and Supplementary References.
