# Publisher Correction: Study of radiative heat transfer in Ångström- and nanometre-sized gaps

**DOI:** 10.1038/ncomms16225

**Published:** 2018-06-25

**Authors:** Longji Cui, Wonho Jeong, Víctor Fernández-Hurtado, Johannes Feist, Francisco J. García-Vidal, Juan Carlos Cuevas, Edgar Meyhofer, Pramod Reddy

Nature Communications 8: Article number: 14479; DOI: 10.1038/ncomms14479 (2017); Published: 02 15 2017; Updated: 06 25 2018

The original HTML version of this Article omitted the article number; it should have been ‘14479’. This has now been corrected in the HTML version of the Article. The PDF version was correct from the time of publication.

