# Supplementary material for: Study of radiative heat transfer in Ångström- and nanometre-sized gaps
Source: Nat Commun. 2017 Feb 15;8:14479. doi: 10.1038/ncomms14479 (PMC5330859; doi:10.1038/ncomms14479)
Supplement: Supplementary Information — Supplementary Figures, Supplementary Notes and Supplementary References. [file ncomms14479-s1.pdf]

SUPPLEMENTARY NOTES

Supplementary Note 1: Fabrication of Scanning Thermal Microscopy Probes

Fabrication of the scanning thermal microscopy (SThM) probes is summarized in Supplementary Fig. 1 and proceeds as follows: (Step 1) To begin, a 500 nm thick low-stress layer of silicon nitride (SiN_x) on a silicon (Si) wafer is deposited by low-pressure chemical vapor deposition (LPCVD). (Step 2) An 8 μm thick layer of low temperature silicon oxide (LTO) is deposited on top of the wafer and is annealed at 1000 °C for 1 hour to reduce the residual stresses of the SiN_x and LTO layers. The LTO layer is then etched to create the sharp probe tip. Subsequently, a 100 nm thick chromium (Cr) layer is sputtered and lithographically patterned by wet etching. This Cr pattern is critical to create the LTO probe tip. (Step 3) The probe tip is created by wet etching in buffered HF (HF: NH_4F = 1 : 5), which takes ~100 minutes. In order to create a sharp tip, the etching status is frequently monitored. (Step 4) A gold (Au) line (Cr/Au: 5/90 nm thick) is lithographically defined by sputtering and wet etching. This Au line forms the first metal layer of the nanoscale Au-Cr thermocouple. Subsequently, a 70 nm thick layer of SiN_x is deposited via plasma enhanced chemical vapor deposition (PECVD) which serves as electrical insulator. (Step 5) A layer of Shipley Microposit S1827 photoresist (6 μm thick) is deposited on the wafer, and the photoresist and PECVD SiN_x are slowly plasma-etched until a very small portion of Au is exposed at the apex of the tip. (Step 6) A Cr line (90 nm thick) is lithographically defined by sputtering and wet etching. This Cr line together with the very small Au extrusion establishes a nanoscale Au-Cr thermocouple at the tip apex. (Step 7) A 70 nm thick PECVD SiN_x is deposited for the electrical insulation. Subsequently, a Au line (Cr/Au: 5/90nm thick) is lithographically defined by sputtering and wet etching. Note that this Au line is the outermost metal layer of the probe. (Step 8) Finally, the SThM probes are released by deep reactive ion etching (DRIE) to create the desired SThM probes. The dimensions of the cantilever are chosen to yield a probe stiffness in excess of 10^4 Nm^{-1}.

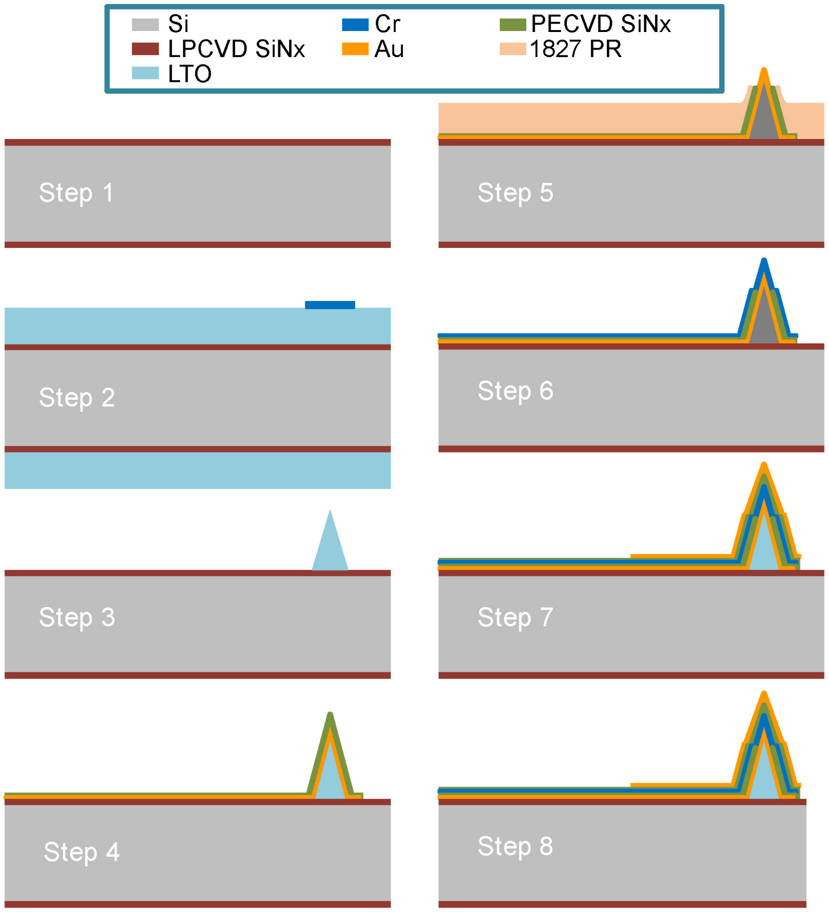

Supplementary Figure 1: Fabrication of the SThM probes (A short description of the fabrication steps is provided in the above section). Not drawn to scale.

Supplementary Note 2: Estimation of the Stiffness of the Scanning Probes via Modeling

The stiffness of the probes was estimated by employing finite element analysis using COMSOL^{TM}. In this computation, we included a 500 µm thick silicon (Si) block, which is the cantilevered portion of our probe, to which a 8 µm tall tip made of silicon oxide (SiO_2) is added as shown in Fig. S2. The values of Young's modulus (E) and Poisson's ratio (v) assumed in these calculations are as follows: Si (E = 170 GPa, v = 0.28), SiO_2 (E = 70 GPa, v = 0.17). Further, in order to estimate the stiffness of the probe, the following boundary conditions were assigned: a 100 nN of either a normal or a shear force was applied at the apex of SiO_2 tip, while the opposite end of the Si block was fixed (see Supplementary Fig. 2). Note that we evaluated three sets of deflections, where the normal deflection (i.e. deflection in the z-direction, see Supplementary Fig.

2) is determined by the cantilever stiffness, whereas the shear deflections (x- or y- direction) are related to the transverse stiffness of the tip. From the computed deflections, the stiffness of our probe was estimated to be ~10700 Nm^{-1} in the normal direction and ~5300 Nm^{-1} in the lateral directions (x and y directions labeled in Supplementary Fig. 2).

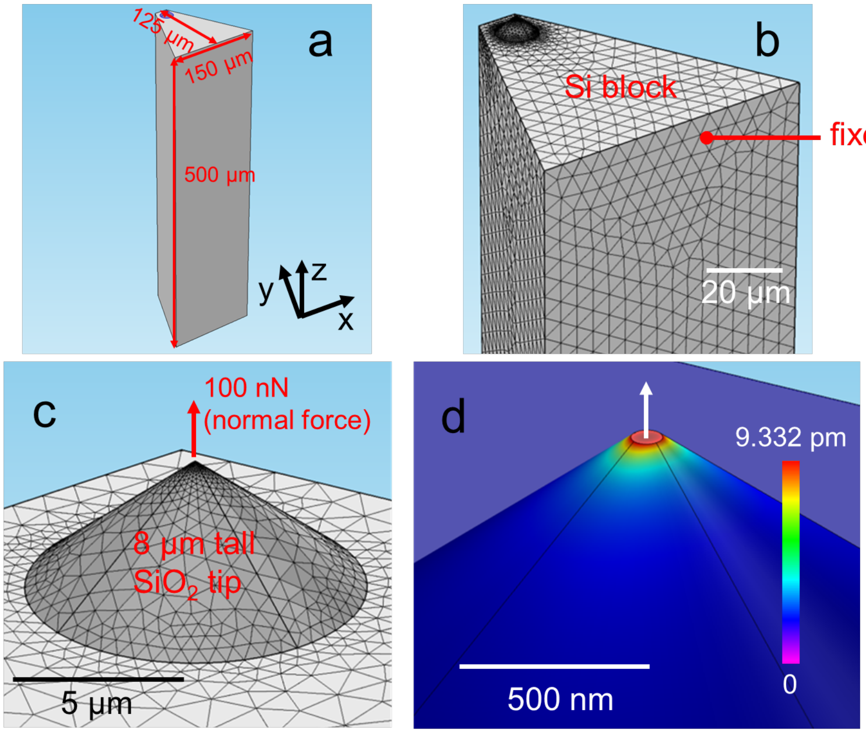

Supplementary Figure 2: Finite element analysis of the scanning probe. (a) Schematic of the probe. (b) & (c) Description of the finite element mesh employed in the calculations. (d) Calculated deflection of the probe in the z-direction in response to a 100 nN normal force.

Supplementary Note 3: Characterization of the Thermal Resistance of Scanning Probes

To characterize the thermal resistance of the SThM probes, we followed an experimental procedure developed by some of us recently[1]. The first step in this process was to determine the heat flux Q into the probe when it contacted a hot surface and measure the temperature increase of the probe (ΔT^{P}) via the embedded thermocouple. The resistance of the probe can thus be found to be $R_{\mathrm{probe}} = \Delta T^{\mathrm{P}} / Q$. To accomplish this procedure, a suspended calorimeter[1] with an integrated Pt resistance heater-thermometer was employed. If an AC current at a frequency ω and amplitude I_{ω} is supplied to the Pt heater, temperature oscillations at a frequency 2ω and

amplitude $T_{2\omega}$ are induced. When the SThM probe was placed in contact with the heated calorimeter (see the inset of Supplementary Fig. 3), an additional conduction path (via the probe) was established resulting in a heat current through the probe. This additional conduction path also reduced the amplitude of temperature oscillations by $\Delta T_{2\omega}$. The heat flux into the probe ($Q_{2\omega}$) can be readily estimated from $Q_{2\omega} = G_{\text{sus}} \Delta T_{2\omega}$, where G_{sus} is the thermal conductance of the suspended calorimeter. By measuring the temperature increase of the probe ($\Delta T_{2\omega}^{\text{p}}$) we obtained the probe resistance as $R_{\text{probe}} = \Delta T_{2\omega}^{\text{p}} / Q_{2\omega}$. In Supplementary Fig. 3, the measured temperature increase of the probe at frequency 2ω is plotted against the heat input into the probe. The slope of the plot gives the probe resistance, which we determined to be $R_{\text{probe}} = 9\times10^{4}\,\text{KW}^{-1}$.

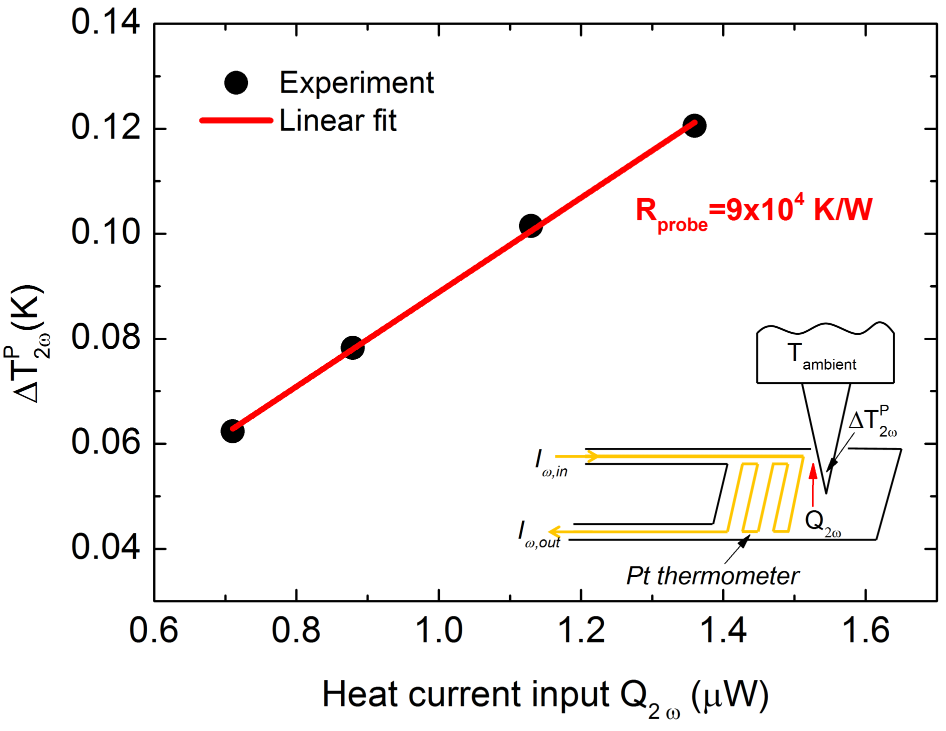

Supplementary Figure 3: Amplitude of temperature oscillations vs. heat current input to the tip. The slope of the line is used to determine the thermal resistance of the probe. Inset shows a schematic of the experiment where the scanning probe is placed in contact with the suspended calorimeter.

Supplementary Note 4: Surface Characterization

The surface topography of template-stripped Au samples and Au-coated scanning probes were characterized by scanning tunneling microscopy (STM) and scanning electron microscopy (SEM), respectively. We note that template-stripped Au surfaces have been widely used in scanning probe microscopy studies due to their high quality in terms of the ultra-small surface roughness.[2] As shown in Supplementary Fig. 4a, the RMS roughness of the Au sample (100 nm thick) obtained from STM studies was found to be <0.1 nm within a scanning area of 150 nm x 150 nm. On the other hand, the probe's (as shown in Supplementary Fig. 4b) surface roughness is found to be much larger (RMS of 2 – 3 nm) than that of the Au sample. This is mostly because that the tip of the scanning probe is a layered structure comprising of metallic and dielectric materials the fabrication (Supplementary Fig. 1) of which involves multiple deposition and etching steps during which the layers tend to become progressively rougher. Since the surface roughness of the Au substrates used in our experiments was substantially smaller than that of the scanning probes, the roughness of the substrates was considered negligible in our computational analysis.

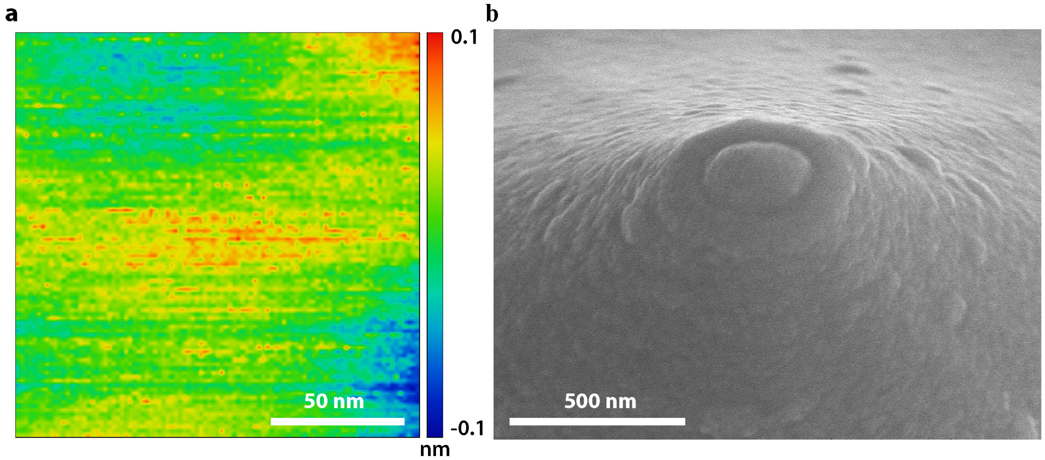

Supplementary Figure 4: Surface topographies of the template-stripped Au sample and the scanning probe. (a) Scanning tunneling microscopy (STM) image of the 100 nm thick template-stripped Au sample. The scanning area is 150 nm by 150 nm. (b) Scanning electron microscope (SEM) image of the scanning probe surface.

Supplementary Note 5: Characterization of the Temperature Drift of the Ambient and the Noise Spectrum of the SThM Probes

The thermoelectric voltage output from the thermocouple embedded in the SThM probe is characterized by noise contributions mainly from Johnson noise and low frequency temperature drift. This noise was quantified by experimentally determining the power spectral density (Supplementary Fig. 5) of the voltage output from the thermocouple using a SR 760 spectrum analyzer (Stanford Research Systems).

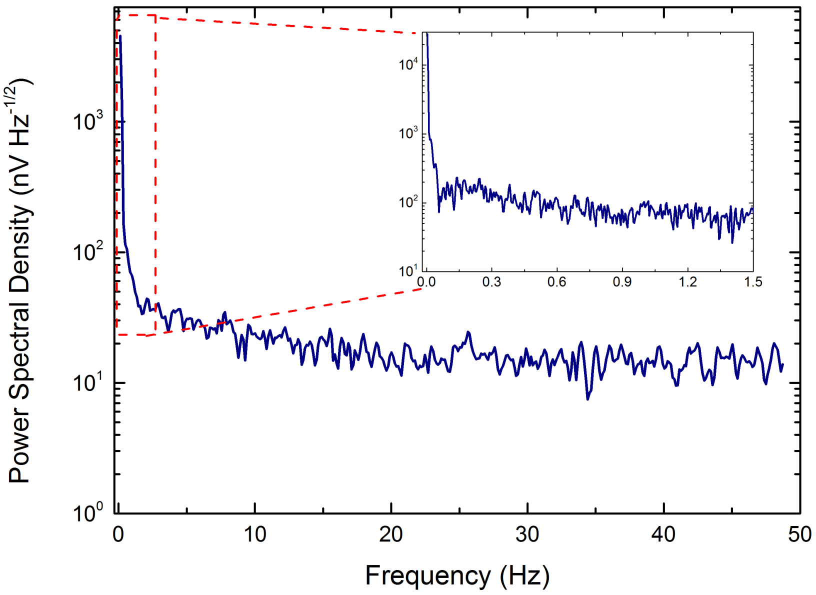

Supplementary Figure 5: Noise characterization of thermoelectric voltage output from the scanning thermal probes. Measured power spectral density (PSD) of the thermoelectric voltage output from the probe for the frequency span from 0 to 50 Hz. The inset shows the PSD for the low frequency (0 to 1.5 Hz) region.

It can be seen that the measured noise power spectral density increases rapidly at low frequencies. The measured PSD at high frequencies agrees reasonably well with the expected Johnson noise (PSD [V/Hz$^{1/2}$]= $\sqrt{4k_BTR}$) and is estimated to be ~10 nVHz$^{-1/2}$ for our scanning probes whose thermocouple resistance is ~5 kΩ. At lower frequencies there are significant contributions due to ambient temperature drift. To demonstrate this point, we recorded the fluctuation of thermal conductance for a period of ~1 hour when the scanning probe was placed at a constant distance of ~100 nm from the substrate (Supplementary Fig. 6). Under these conditions the thermal

conductance between the tip and the sample is expected to be invariant with time. It can be seen that there is an apparent thermal conductance change of ~15 nWK^{-1} (peak-to-peak), similar to the noise level shown in Fig. 4 of the main manuscript which is likely due to temperature drift of a few 10s of mK. Furthermore, a comparison of the measured frequency-dependent noise spectral densities in Supplementary Fig. 6 and Fig. 4, suggests that the low frequency noise present in the data of Fig. 4 is most likely due to the temperature drift of the ambient.

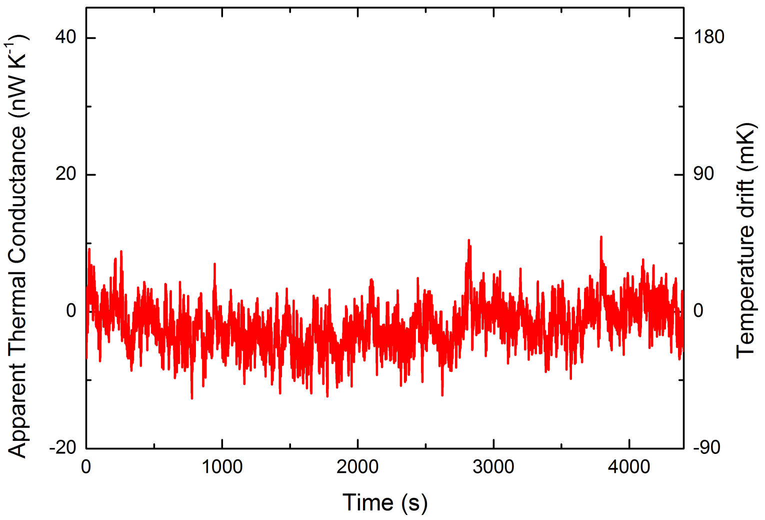

Supplementary Figure 6: Fluctuations in temperature and radiative thermal conductances. Temperature drift of the scanning probe or the ambient temperature leads to fluctuations of the thermoelectric voltage output of the scanning probe, which in turn manifests itself as apparent thermal conductance fluctuations. The above data, which illustrate the level of noise in the thermal conductance data, were obtained from an experiment where the probe and the sample were separated by 100 nm and a temperature differential of 40 K was applied.

Supplementary Note 6: Probe Diameter Characterization in "Controlled Crashing" Experiment

In the "controlled crashing" experiments the tip of the scanning probe is indented into the substrate by a very short distance (a few nanometers at most). To verify that there is no significant change in the tip diameter during this process we obtained SEM images for a new probe and of a probe after the crashing experiment. As shown in Supplementary Fig. 7, we found no gross/observable change in the tip shape or curvature.

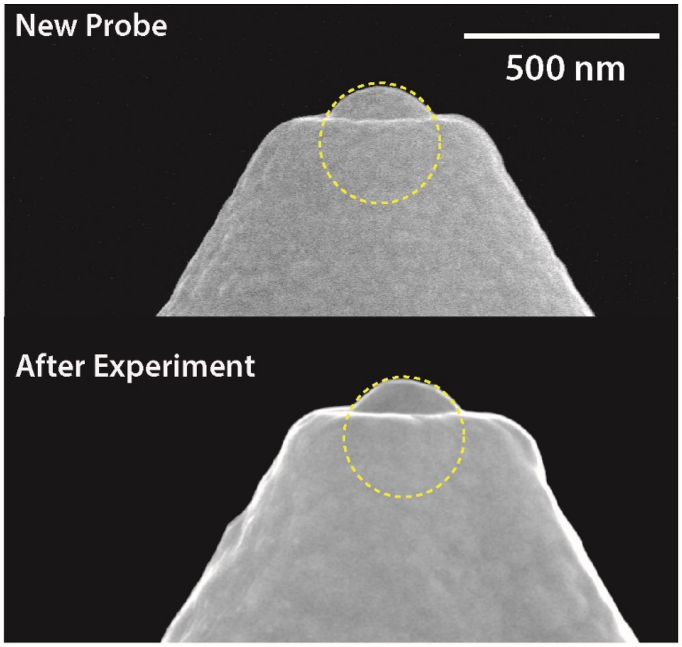

Supplementary Figure 7: SEM images of a pristine probe and a probe subjected to "controlled" crashing. Analysis of the SEM images of the tips of scanning thermal probes before and after subjecting to controlled crashing suggests that there is nor observable difference in the tip geometry. The diameter of the dashed circles is ~300 nm.

Supplementary Note 7: Determination of the Gap-size

Unambiguous determination of the gap-size between the scanning probe and the sample is key to successful interpretation of the data obtained in our experiments. We estimated the minimum gap-size from the conductance vs. gap-size curve (Fig. 2) by extrapolating the curve to a conductance of $1G_0$ (corresponding to a single-atom Au-Au contact) and estimating the additional distance the probe would have to be moved to achieve this conductance. This additional distance is an indicator of the minimal gap-size in our experiments. Since the measured tunneling barrier was found to be ~1 to 2.5 eV, the extrapolated gap-size was estimated to be ~1.3 to 2.2 Å.

This estimate was also experimentally validated by displacing our probes (in control experiments) until the conductance was increased to $1G_0$ from $0.1G_0$. Data from such experiments is shown in Supplementary Fig. 8 and it can be seen that an additional displacement of ~1 to 2.5 Å is required to increase the conductance to $1G_0$.

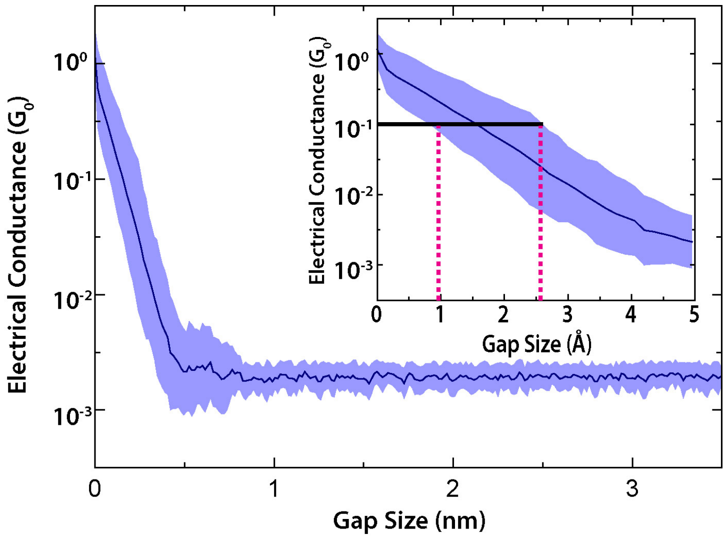

Supplementary Figure 8: Determination of the gap-size from electrical conductance measurements. The gap-size is defined to be zero ("contact") when the electrical conductance is equal to $1G_0$, indicating the formation of an atomic contact. Measured electrical conductances (in units of the quantum of conductance) between the Au-coated scanning probe and the Au sample are plotted as a function of the gap-size. The electrical conductance at the smallest gap-sizes (below 5 Å) is shown in the inset to facilitate visualization. From this plot we determine that the mean gap size at an electrical conductance of 0.1Go is ~1.6 Å, ranging from 1 – 2.5 Å.

Supplementary references:

1. Kim, K., *et al.* Quantification of thermal and contact resistances of scanning thermal probes. *Appl. Phys. Lett.* **105**, 203107(2014).

2. Hegner, M., Wagner, P., Semenza, G.. Ultralarge atomically flat template-stripped Au surfaces for scanning probe microscopy. *Surf. Sci.* **291**, 39-46 (1993).
